# MicroRNA‐150 and its target ETS‐domain transcription factor 1 contribute to inflammation in diabetic photoreceptors

**DOI:** 10.1111/jcmm.17012

**Published:** 2021-10-26

**Authors:** Fei Yu, Michael L. Ko, Gladys Y.‐P. Ko

**Affiliations:** ^1^ Department of Veterinary Integrative Biosciences College of Veterinary Medicine and Biomedical Sciences Texas A&M University College Station Texas USA; ^2^ Department of Biology Division of Natural and Physical Sciences Blinn College Bryan Texas USA; ^3^ Texas A&M Institute for Neuroscience Texas A&M University College Station Texas USA

**Keywords:** diabetes, diabetic retinopathy, inflammation, microRNA, obesity, photoreceptor

## Abstract

Obesity‐associated type 2 diabetes (T2D) is on the rise in the United States due to the obesity epidemic, and 60% of T2D patients develop diabetic retinopathy (DR) in their lifetime. Chronic inflammation is a hallmark of obesity and T2D and a well‐accepted major contributor to DR, and retinal photoreceptors are a major source of intraocular inflammation and directly contribute to vascular abnormalities in diabetes. However, how diabetic insults cause photoreceptor inflammation is not well known. In this study, we used a high‐fat diet (HFD)‐induced T2D mouse model and cultured photoreceptors treated with palmitic acid (PA) to decipher major players that mediate high‐fat‐induced photoreceptor inflammation. We found that PA‐elicited microRNA‐150 (miR‐150) decreases with a consistent upregulation of ETS‐domain transcription factor 1 (*Elk1*), a downstream target of miR‐150, in PA‐elicited photoreceptor inflammation. We compared wild‐type (WT) and miR‐150 null (miR‐150^−/−^) mice fed with an HFD and found that deletion of miR‐150 exacerbated HFD‐induced photoreceptor inflammation in conjunction with upregulated ELK1. We further delineated the critical cellular localization of phosphorylated ELK1 at serine 383 (pELK1_S383_) and found that decreased miR‐150 exacerbated the T2D‐induced inflammation in photoreceptors by upregulating ELK1 and pELK1_S383_, and knockdown of ELK1 alleviated PA‐elicited photoreceptor inflammation.

## 1 INTRODUCTION

The incidence of diabetes is projected to rise to 33% of the US population by 2050 owing to the obesity epidemic,^1^ of which 95% of diabetic patients will have type 2 diabetes (T2D).^2^ More than 85% of T2D patients have diabetes‐related eye disorders, and 60% develop diabetic retinopathy (DR), the leading cause of blindness in US adults age 20–74.^2^, ^3^ While anti‐vascular endothelial growth factor (VEGF) treatments significantly improve the outcomes of DR, nearly 30% of patients do not respond to anti‐VEGFs,^4^, ^5^ making the development of new treatment strategies imperative. Chronic inflammation is a hallmark of obesity and T2D^6^, ^7^ and a well‐accepted major contributor to DR,^8^, ^9^ but numerous studies have indicated that intraocular rather than systemic inflammation is more closely associated with the vascular complications in DR.^10^, ^11^ Interestingly, diabetic patients who also have retinitis pigmentosa (RP), a congenital blindness with initial degeneration of rod photoreceptors, rarely develop DR, even though they have other non‐retinal diabetic vascular complications.^12^, ^13^ Thus, there is a clear inverse correlation between RP and DR.^12^, ^13^ In mice, genetic deletion of rod photoreceptors or pharmacological inhibition of photoreceptors reduces retinal inflammation and alleviates progression of DR.^14^, ^15^ Therefore, retinal photoreceptors are a major source of intraocular inflammation and directly contribute to vascular abnormalities in diabetes. However, how photoreceptors contribute to intraocular inflammation and vascular complications under T2D is still not well elucidated.

MicroRNAs (miRs) are small non‐coding RNAs containing 22~25 nucleotides, and their seed sequences (nucleotides 2–8) pair with the complementary 3’ untranslated region (UTR) of their target mRNAs and mediate the translational inhibition or degradation of mRNAs.^16^ MicroRNAs represent a set of modulators that regulate metabolism, inflammation and angiogenesis,^17^, ^18^, ^19^ and changes of various miR levels observed in diabetic patients have been linked to the pathogenesis of DR.^20^, ^21^ Among them, there is a strong correlation between miR‐150 downregulation and patients with diabetes and DR. Retinal miR‐150 is decreased in the eyes under ischaemic insults^22^ and in patients with proliferative DR.^23^ Serum miR‐150 is decreased in patients with obesity,^24^ T1D^25^, ^26^ or T2D,^27^, ^28^ in association with increased inflammation and upregulation of angiogenic factors. We and others have reported that miR‐150 is significantly decreased in the blood, heart, and retina in animals with high‐fat diet (HFD)‐induced T2D^29^ or streptozotocin (STZ)‐induced T1D.^20^, ^30^ Deletion of miR‐150 in mice (miR‐150^−/−^) further exacerbates obesity‐associated T2D and T2DR compared with the wild‐type (WT) mice, including significantly elevated systemic insulin resistance, glucose intolerance, inflammation,^19^ worsen retinal light responses,^29^ and more severe retinal microvascular degeneration and leakage.^23^ Thus, downregulation of miR‐150 correlates with the progression of diabetes and DR.

In addition, miR‐150 is an intrinsic suppressor of inflammation.^19^ Overexpression of miR‐150 downregulates TNF‐α and nuclear factor kappa B (NF‐ĸB) induced by lipopolysaccharide (LPS) in endothelial cells.^31^ Deletion of miR‐150 (miR‐150^−/−^) exacerbates the increase of TNF‐α and IL‐1β in mice with an HFD‐induced T2D.^19^ We previously showed that miR‐150^−/−^‐HFD mice have more severe inflammation in photoreceptors and exacerbated vascular degeneration compared with the WT‐HFD mice.^23^ Overexpression of miR‐150 protects the retinal vasculature from degeneration induced by oxygen‐induced retinopathy (OIR), a well‐established model for pathological angiogenesis.^32^ Moreover, overexpressing miR‐150 restores endothelial cell functions including proliferation and migration.^33^ Therefore, miR‐150 could restrain the development of DR by mitigating inflammation in the neural retina especially in photoreceptors. However, how miR‐150 and its downstream targets contribute to diabetes‐induced inflammation in photoreceptors remains unclear.

The biological processes mediated by miRs and their targets are often tissue‐ and cell‐type‐dependent.^16^ Previously, Dr. B. Zhou's laboratory screened the top 30 predicted target genes of miR‐150 by combining computational analyses, transcriptome profiles, and reporter assays,^19^, ^34^, ^35^ and identified new *bona fide* targets that are pro‐inflammatory.^19^ Among them, ETS‐domain transcription factor 1 (*Elk1*), eukaryotic translation termination factor 1 (*Etf1*), early growth response 1 (*Egr1*) and MYB proto‐oncogene (c‐*Myb*) are expressed in photoreceptors and retinal endothelial cells. In this study, we used a functional assay with cultured photoreceptors treated with palmitic acid (PA) to generate a high‐fat environment for cells. We found that PA elicited decreased miR‐150 with a consistent upregulation of *Elk1* but not others, so we focussed on *Elk1* and its associated signalling in promoting retinal inflammation in T2DR with *in vitro* and *in vivo* assays. We compared WT and miR‐150^−/−^ mice fed with an HFD and determined the role of miR‐150 and *Elk1* in mediating inflammation in photoreceptors under T2D. We further used cultured 661W cells, a mouse photoreceptor cell line,^36^ to decipher the relationship between miR‐150, *Elk1*, ELK1 and inflammation in photoreceptors. We delineated the critical cellular localization of phosphorylated ELK1 at serine 383 (pELK1_S383_) and HFD‐ or PA‐associated inflammation in photoreceptors. Our data indicate that decreased miR‐150 exacerbates the T2D‐induced inflammation in photoreceptors by upregulating ELK1 and pELK1_S383_, and knocking down *ELK1* alleviates the inflammation and reduces pELK1_S383_.

## 2 MATERIALS AND METHODS

### 2.1 Animals

Four‐week‐old male C57BL/6J mice (wild type, WT) were purchased from the Jackson Laboratory. B6(C)‐Mir150^tm1Rsky^/J (miR‐150^−/−^) mice were originally purchased from the Jackson Laboratory, and a colony was bred and maintained at Texas A&M University. Only male mice were used in this study. All animal experiments were approved by the Institutional Animal Care and Use Committee of Texas A&M University and were performed in compliance with the Association for Research in Vision and Ophthalmology (ARVO) Statement for the Use of Animals in Ophthalmic and Vision Research. Mice were housed under temperature and humidity‐controlled conditions with 12:12 h of light‐dark cycles. All mice were given food and water *ad libitum*. At 5 weeks of age (body weight, 20 g), mice were fed a high‐fat diet (HFD; 60% fat calories, 20% protein calories and 20% carbohydrates calories; #D12492; Research Diets) or a control diet (standard laboratory chow; 10% fat calories, 20% protein calories and 70% carbohydrates calories; #D12450J; Research Diets) for up to 24 weeks. Bodyweight and food intake were measured weekly. Non‐fasting blood glucose levels and glucose tolerance were measured monthly by taking blood from the tail vein. Glucose levels were measured using a Clarity BG1000 blood glucose monitoring system (Clarity Diagnostics).

### 2.2 Cell culture

The 661W cells^36^ were originally obtained from Dr. Al‐Ubaidi (University of Houston) and cultured in Dulbecco's modified Eagle medium (DMEM; #12‐614Q, Lonza) containing 10% foetal bovine serum (FBS; #S11550, R&D Systems), 2 mM Glutamax (#35050‐061, Gibco/ThermoFisher), 100 μg/ml penicillin and 100 μg/ml streptomycin (#15140‐148, Gibco/ThermoFisher), and 1 mM sodium pyruvate (#S8636, Sigma) at 37°C and 5% CO_2_. The 661W cells were treated with 100 µM palmitic acid (PA, #P5585‐10G, Sigma) dissolved in 10% bovine serum albumin (BSA; #A6003‐25G, Sigma) or an equal volume of 10% BSA (vehicle control) for various times as indicated.

### 2.3 Lipofectamine transfection

Transfection was conducted using the Lipofectamine 3000 kit (#L3000015, Invitrogen/ThermoFisher) according to the manufacturer's instruction. Briefly, the 661W cells were seeded at 30% confluency and allowed to grow for 24 h to reach 50% confluency. For Western blot and qPCR, cells were seeded in 6‐well plates and transfected with 30 pmol/well microRNA (miRNA)/siRNA. For Terminal deoxynucleotidyl transferase dUTP nick end labelling (TUNEL) and immunofluorescent staining, the cells were seeded on 12 mm circular coverslips in 24‐well plates and transfected with 10 pmol/well miRNA/siRNA. After the first exchange to normal culture medium, some cultures were immediately treated with PA or BSA for various hours. The following miRNAs / siRNAs were used in this study: miRNA negative control (#4464058, ThermoFisher), miR‐150 mimic (Assay MC10070, #4464066, ThermoFisher), miR‐150 inhibitor (Assay MH10070, #4464084, ThermoFisher), siRNA‐negative control (#AM4613, ThermoFisher) and Elk1 siRNA (Assay 261017, #AM16708, ThermoFisher).

### 2.4 Quantitative real‐time RT‐PCR (qPCR)

After cells were collected, total RNA from each sample was prepared by using a commercially available purification kit (miRNeasy mini kit; #217004, Qiagen). From each sample, 500 ng of total RNA was used to quantify miR‐150 or mRNAs by qPCR using a TaqMan MicroRNA Reverse Transcription Kit (#4366596, ThermoFisher) and SYBR green supermix ROX (#95055‐500, QuantaBio) with a CFX Connect Real‐Time PCR Detection System (Bio‐Rad). The primers (Bioneer) of *Elk1* (Forward 5’‐GCC GGG CCT TGC GGT ACT ACT ATG A‐3’, Reverse 5’‐GGG TAG GAC ACA AAC TTG TAG AC‐3’), *Etf1* (Forward 5’‐TTG AAC CTT TCA AAC CAA TTA ATA C‐3’, Reverse 5’‐CAG TGA ATT TGT GCA GGA CTT CTC T‐3’), *Egr1* (Forward 5’‐GCA ACG GGG CTC CCC AGT TCC TCG G‐3’, Reverse 5’‐AAG CGG CCA GTA TAG GTG ATG GG‐3’), *c*‐*Myb* (Forward 5’‐CCA GCA AGG TGC ATG ATC GTC CAC C‐3’, Reverse 5’‐AGA ATT CAA AAC TGC TGA GAT CAC A‐3’) and *β*‐*actin* (Forward 5’‐CAA CGG CTC CGG CAT GTG C‐3’, Reverse 5’‐GTA CAT GGC TGG GGT GTT GAA GGT C‐3’) were used in this study. The hsa‐miR‐150 assay (Assay 000473, #4440887, ThermoFisher) and U6 snRNA assay (Assay 001973, #4440887, ThermoFisher) were used to test the levels of miR‐150.

For each individual experiment, a standard curve was generated with known quantities of RNAs loaded in curved dilutions (ie 2x, 1x, 1/2, 1/4, 1/8, 1/16 and 1/32). The cycle values, corresponding to the log values of the standard curve quantities, were used to generate a linear regression formula. The amplification efficiency of the qPCR reactions (90%–100%) was calculated using the standard curve. The quantification of sample RNA was calculated by the 2^(−ΔΔCt)^ method^37^ using U6 (for miR‐150) or *β*‐*actin* (for other genes) as the internal control.

### 2.5 Western blot

Samples for Western blots were collected, prepared and analysed as described previously.^38^ Briefly, 661W cells were harvested and lysed in a Tris lysis buffer (in mM): 50 Tris, 1 EGTA, 150 NaCl, 1% Triton X‐100, 1% β‐mercaptoethanol, 50 NaF, and 1 Na_3_VO_4_, pH 7.5. Samples were separated on 10% sodium dodecyl sulphate‐polyacrylamide gels by electrophoresis and transferred to nitrocellulose membranes. The membranes were blocked in 3% BSA in Tris‐buffered saline, Tween 20 (TBST) at room temperature for 1 h and incubated in primary antibodies overnight at 4°C. After washing with TBST, the membranes were incubated in anti‐rabbit IgG HRP‐linked secondary antibody (1:1000, #7074S, Cell Signaling) at room temperature for 1 h. The blots were visualized using Super Signal West Pico/Femto chemiluminescent substrate (#34078 / #34096, ThemoFisher). Band intensities were quantified using ImageJ (NIH). The primary antibodies used in this study were as follows: anti‐ELK1 (1:500, #9182S, Cell Signaling), anti‐phospho NFĸB P65 (1:1000, #3033, Cell Signaling), anti‐NFĸB P65 (1:1000, #8242S, Cell Signaling) and anti‐β‐actin (1:2000, #8457L, Cell Signaling). The band intensities of ELK1 were normalized to those of β‐actin. The band intensities of phosphorylated NFĸB P65 (pP65) were normalized to those of total NFĸB P65 (P65) and β‐actin.

### 2.6 Immunofluorescent staining (retina and cultured cells)

Mouse eyes were collected, fixed with 4% paraformaldehyde and processed for paraffin‐embedded sectioning after 24 weeks of the diet regimen. Paraffin sections (4 μm) of mouse eyes from all four experimental groups were mounted on the same glass slide. The retina sections were deparaffinized by heating at 57°C followed by washing with xylene and serial dilutions of ethanol. The antigen retrieval for retinal sections was carried out in a sodium citrate buffer (10 mM sodium citrate, 0.05% Tween 20, pH 6.0) at 80°C for 1 h. The 661 cells cultured on coverslips were fixed with 4% paraformaldehyde at room temperature for 1 h and permeabilized with 0.1% Triton X‐100 in 0.1% sodium citrate at 4°C for 10 min.

Eye sections or coverslips were then blocked with 10% goat serum in phosphate‐buffered saline (PBS) for 2 h at room temperature and incubated with primary antibodies overnight at 4°C. After washing with PBS, sections or coverslips were incubated with secondary antibodies for 2 h at room temperature and mounted with ProLong Gold antifade mountant with 4′,6′‐diamidino‐2‐phenylindole (DAPI) (#P36935, ThermoFisher). Images were obtained using a Zeiss Axiovert 200 M microscope (Carl Zeiss AG). All fluorescent images were taken under identical settings including light intensity, exposure time and magnification.^23^, ^38^

The fluorescent intensity was measured in the inner and outer segments of photoreceptors (IS/OS) and in the outer nuclear layer (ONL) for mouse retina sections or in the nuclear and cytoplasmic areas of 661W cells using ImageJ (NIH). DAPI was used to identify the nuclear regions of the cells. The intensity of pELK1 in the cytoplasm was measured at the processes of photoreceptors that are 10 µm from the nucleus. The intensity of pELK1 in the nucleus was measured within the DAPI‐stained area. We analysed 10–15 regions for each culture well. The fluorescent intensities of pP65 in the IS/OS or pELK1 in the IS/OS and ONL were measured from 5 to 10 regions and subtracted by the background intensity for each retinal section.

The following primary antibodies were used as follows: anti‐phospho NFĸB P65 (1:50, #3033, Cell Signaling) and anti‐phospho‐ELK1(S383; 1:50, #ab218133, Abcam). The following secondary antibodies were used as follows: goat anti‐rabbit IgG (Alexa Fluor 488; 1:50, #ab150077, Abcam) and goat anti‐rabbit IgG (Alexa Fluor 568; 1:50, # ab175471, Abcam).

### 2.7 Statistical analysis

All data are presented as mean ± standard error of the mean (SEM). Student’s *t* test or one‐way analysis of variance (ANOVA) followed by Tukey's *post hoc* tests were used for statistical analyses among groups. Throughout, *p *< 0.05 was regarded as significant. Origin 9.0 (OriginLab) was used for statistical analyses.

## 3 RESULTS

### 3.1 Deletion of miR‐150 (miR‐150^−/−^) exacerbates inflammation in the obesity‐associated T2D retina

**FIGURE 1:**
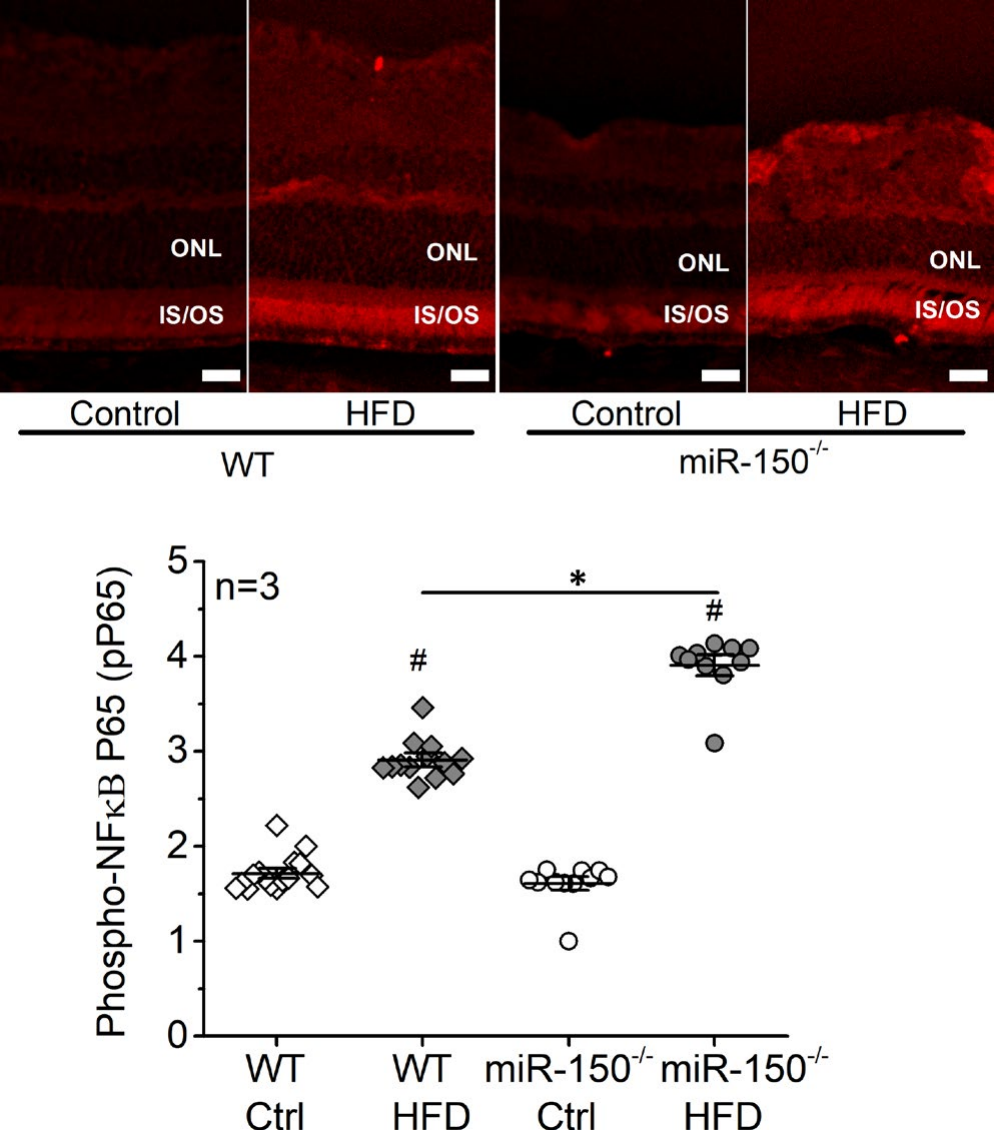
Deletion of miR‐150 (miR‐150^−/−^) exacerbates inflammation in the obesity‐associated T2D retina. Wild‐type (WT) and miR‐150^−/−^ mice were fed a normal chow (Ctrl) or high‐fat diet (HFD) for 6 months. Retinal sections were immunostained with phosphorylated NFĸB P65 (pP65; red). The fluorescent intensities in the retinal outer nuclear layer (ONL) were quantified using ImageJ. WT‐Ctrl: open diamond; WT‐HFD: dark diamond; miR‐150^−/−^‐Ctrl: open circle; miR‐150^−/−^‐HFD: dark circle. # indicates a statistical significance when compared to WT‐Ctrl and miR‐150^−/−^‐Ctrl; * indicates a statistical significance between the two HFD groups. *N* = 3 mice; *p *< 0.05, one‐way ANOVA. Scale bar: 20 µm

Inflammation is a major culprit in the pathogenesis of DR.^9^, ^39^ We previously showed that mice fed with an HFD develop obesity‐associated T2D, in which inflammation is detected in the vitreous and neural retina, and phosphorylated NFĸB P65 (pP65; a biomarker for inflammation) but not the total P65 is significantly increased in the whole retina.^40^, ^41^ Using immunostaining, we found that deletion of miR‐150 (miR‐150^−/−^) further exacerbates retinal inflammation in obesity‐associated T2DR^23^ especially in the photoreceptors (Figure 1). The HFD‐T2D mice (WT and miR‐150^−/−^) had increased pP65 in the outer nuclear layer (ONL), as well as the inner and outer segments of photoreceptors (IS/OS), compared to the mice fed with normal chow. In addition, the miR‐150^−/−^‐HFD mice had further increased pP65 than the WT‐HFD mice (Figure 1). The results demonstrate that miR‐150 knockout exacerbates the HFD‐induced retinal inflammation, especially in photoreceptors.

### 3.2 MiR‐150 knockout exacerbates palmitic acid (PA)‐elicited inflammation in cultured 661W cells

**FIGURE 2:**
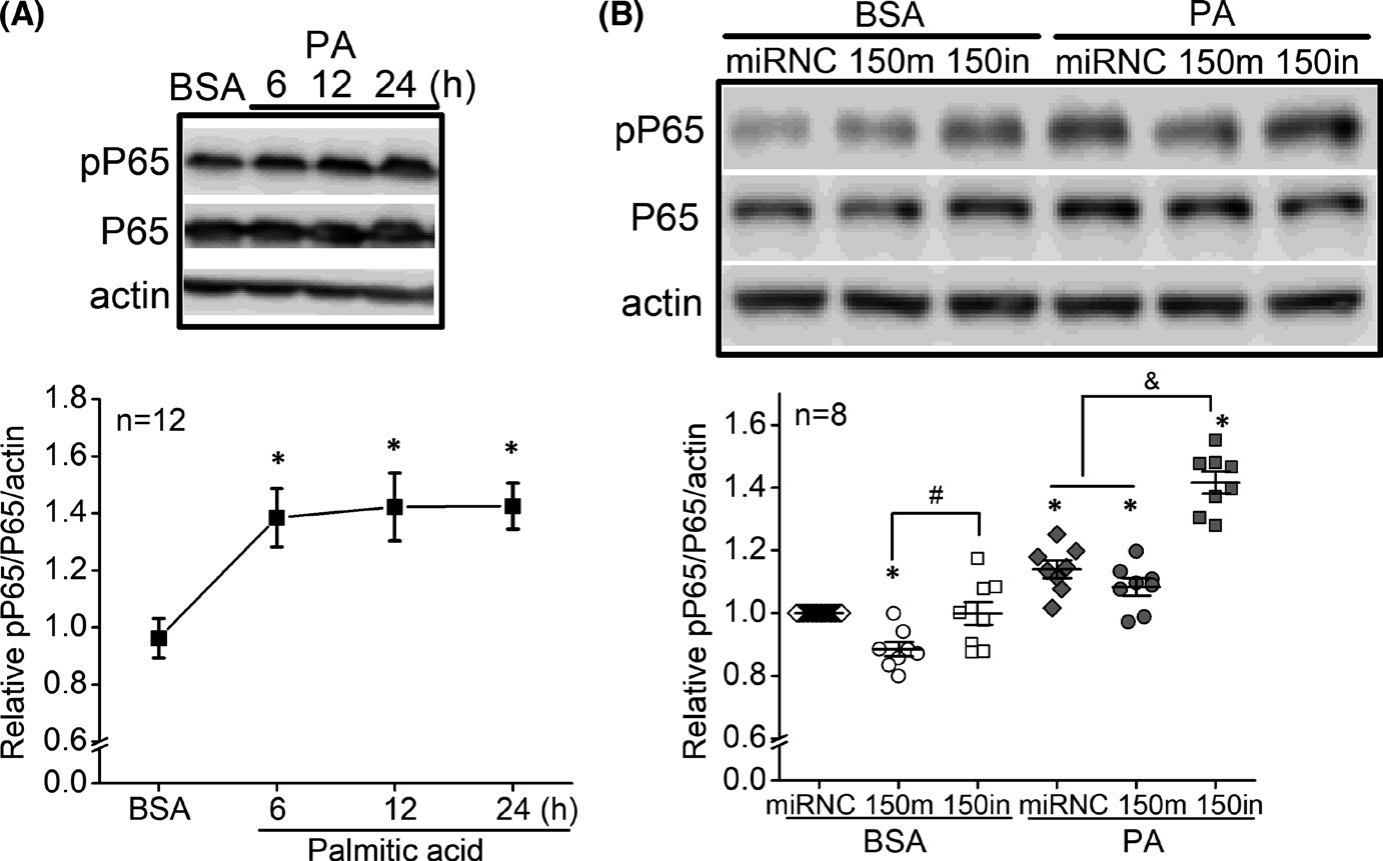
MiR‐150 knockout exacerbates PA‐elicited inflammation in cultured 661W cells. (A) 661W cells were treated with 100 µM PA or BSA (vehicle) for 6–24 h. The protein levels of P65, pP65 and actin (loading control) were determined by Western blots. * indicates a statistical significance when compared to BSA. *N* = 12 experimental trials. *p *< 0.05, one‐way ANOVA. (B) The 661W cells were first transfected with a microRNA‐negative control (miRNC), miR‐150 mimic (150m), or miR‐150 inhibitor (150in) and then treated with BSA or PA for 24 h. The protein levels of P65, pP65 and actin (loading control) were determined by Western blots. miRNC‐BSA: open diamond; 150m‐BSA: open circle; 150in‐BSA: open square; miRNC‐PA: dark diamond; 150m‐PA: dark circle; 150in‐PA: dark square. * indicates statistical significance when comparing miRNC‐BSA with miRNC‐BSA, and comparing PA‐treated cells to BSA‐treated cells. # indicates statistical difference between 150m‐BSA and 150in‐BSA. & indicates a statistical difference when comparing150in‐PA with miRNC‐PA and 150m‐PA. *p *< 0.05, one‐way ANOVA

As miR‐150 is decreased in the blood and retina in diabetic patients^23^, ^25^, ^26^, ^42^ and animals with streptozotocin‐induced T1D or HFD‐associated T2D,^23^ there is a correlation between decreased miR‐150 and diabetes. We next tested whether decreased miR‐150 directly triggered inflammatory responses, or miR‐150 is a medium linking diabetic insults and inflammation using cultured 661W cells.^36^ The 661W cells were originally derived from a mouse retinal tumour and characterized as a cone‐photoreceptor cell line for expressing opsins, transducin and arrestin,^36^ and they are widely used in photoreceptor research. We found that cultured cells treated with palmitic acid (PA, 100 µM) had significantly increased levels of pP65, indicating that PA triggered inflammatory responses in 661W cells (Figure 2A). We transfected cells with miR‐150 mimics (150m), miR‐150 inhibitor (anti‐miR‐150 antagomir; 150in) or a nonspecific miR as a negative control (miRNC). While cells transfected with miR‐150 mimics had decreased pP65, knocking down miR‐150 (150in) in cultured cells did not alter the pP65 level (Figure 2B). This indicates that miR‐150 has intrinsic anti‐inflammatory properties as previously reported,^19^, ^31^ but decreasing miR‐150 alone does not cause inflammation. However, if transfected cells were treated with PA for 24 h regardless of whether they were transfected with 150m or 150in, they all had significantly increased pP65 compared to cells treated with the vehicle control (BSA). Cells with miR‐150 knockdown (150in) had further exacerbated increases of PA‐elicited pP65 compared to cells transfected with miRNC and miR‐150 mimics (150m; Figure 2B). These results show that miR‐150 knockdown aggravates the PA‐elicited inflammation in cultured 661W cells while overexpressing miR‐150 is not sufficient to overturn PA‐elicited inflammation. Thus, diabetic insults elicit inflammation responses, and decreased miR‐150 will further aggravate the retinal inflammation.

### 3.3 *Elk1*, but not *c*‐*Myb*, *Etf1* or *Egr1*, is the direct target of miR‐150 in response to PA treatments in 661W cells

There are several *bona fide* targets of miR‐150 known to be pro‐inflammatory including *c*‐*Myb*, *Etf1*, *Egr1* and *Elk1*.^19^ In cultured adipose B lymphocytes, lipopolysaccharides (LPS) induces inflammatory responses that correspond with decreased miR‐150 and upregulated *c*‐*Myb*, *Etf1*, *Egr1* and *Elk1*, and knockdown of miR‐150 further increases the expression of these genes and escalates LPS‐elicited inflammation.^19^ Because miRs and their downstream targets have tissue‐ and cell type‐specific bioactivities,^16^, ^43^ we set forth to determine the downstream target(s) of miR‐150 responsible for diabetes‐associated inflammation in retinal photoreceptors.

**FIGURE 3:**
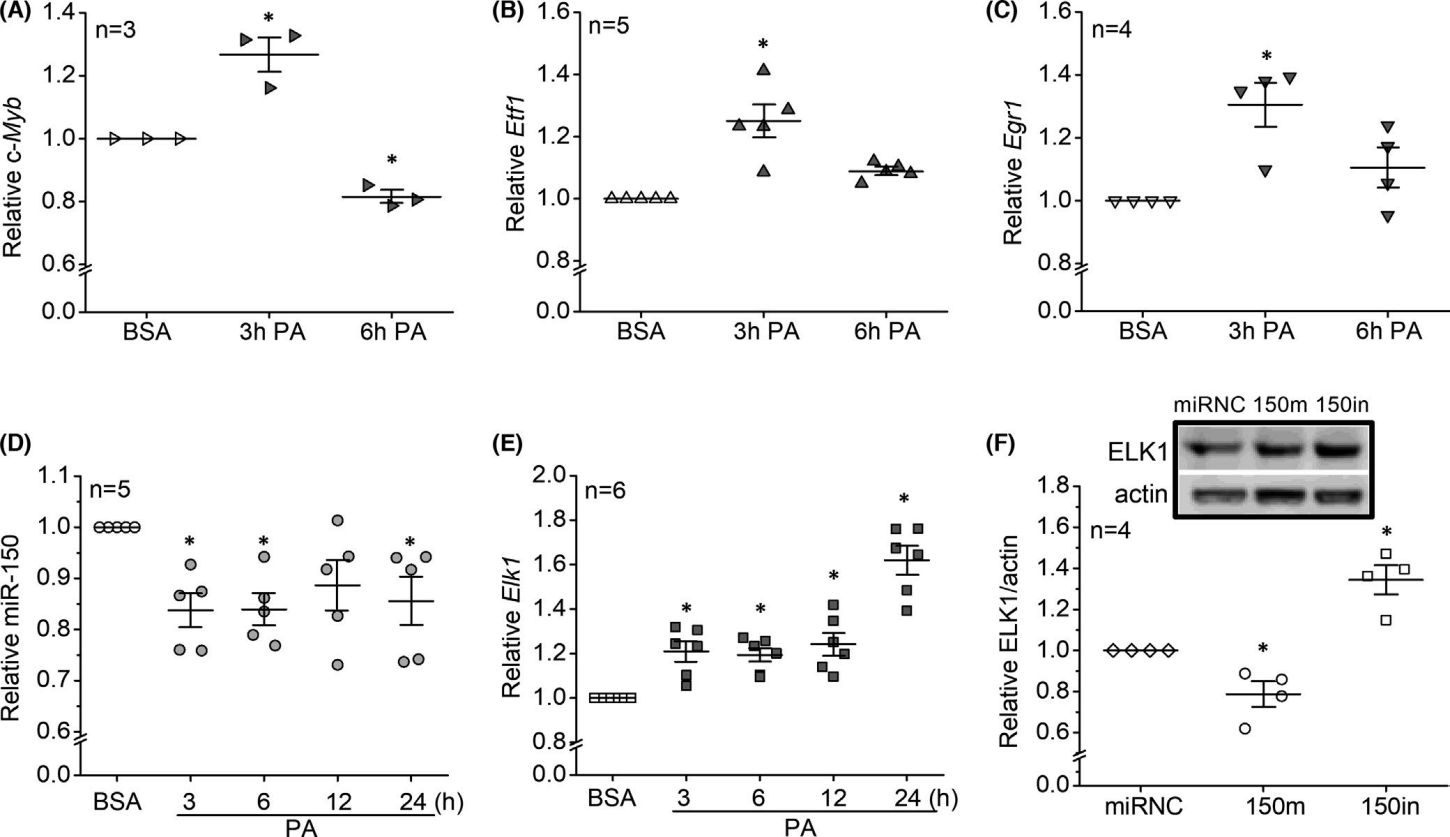
*Elk1*, but not *c*‐*Myb*, *Etf1*, or *Egr1*, is the direct target of miR‐150 in responses to PA treatments in 661W cells. (A–E) The 661W cells were treated with BSA (vehicle) or 100 µM PA (PA) for various times. The mRNA or miR levels of c‐*Myb* (A), *Elf1* (B), *Egr1* (C), miR‐150 (D) and *Elk1* (E) were determined by qPCR. * indicates a statistical difference when compared to the BSA treatment. (F) 661W cells were transfected with a microRNA‐negative control (miRNC), miR‐150 mimic (150m) or miR‐150 inhibitor (150in). The ELK1 levels were measured using Western blots. * indicates a statistical difference when compared to the miRNC. *p *< 0.05, one‐way ANOVA

Using cultured 661W cells treated with 100 µM PA, we found that at 3 h these cells had transient increases of *c*‐*Myb*, *Etf1* and *Egr1* (Figure 3A–C), even though miR‐150 was decreased consistently in the presence of PA (3–24 h; Figure 3D). *Elk1* was the only downstream target that was consistently increased in the presence of PA (3–24 h; Figure 3E). In addition, ELK1 protein was decreased in cells transfected with a miR‐150 mimic (150m) but increased in cells transfected with the miR‐150 inhibitor (150in; Figure 3F). These results confirmed that *Elk1* is a direct target of miR‐150 in 661W cells in responding to PA.

### 3.4 Knocking down *Elk1* alleviates PA‐induced inflammation in 661W cells

**FIGURE 4:**
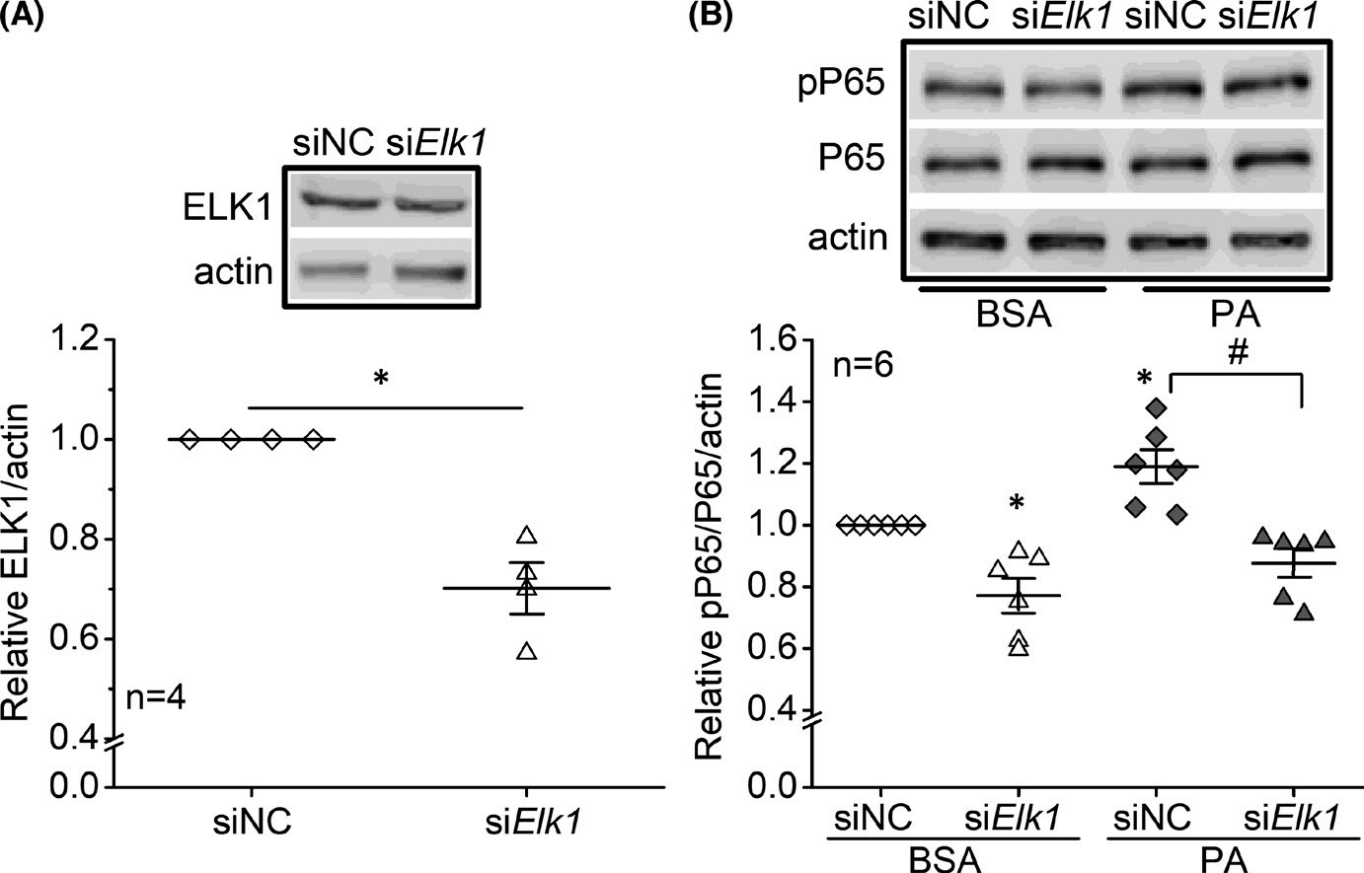
Knocking down *Elk1* alleviates PA‐induced inflammation in 661W cells. The 661W cells were transfected with siRNA‐negative control (siNC) or *Elk1* siRNA (si*Elk1*). (A) After transfections, cells were harvested, and the ELK1 protein levels were measured by Western blots. * indicates a statistical significance between siNC (open diamond) and si*Elk1* (open triangle) groups. *p *< 0.05, Student's *t* test. (B) After transfections, 661W cells were treated with BSA or 100 µM PA (PA) for 24 h. Cells were harvested and the protein levels of P65, pP65 and actin (loading control) were measured using Western blots. siNC‐BSA: open diamond; si*Elk1*‐BSA: open triangle; siNC‐PA: dark diamond; si*Elk1*‐PA: dark triangle. * indicates a statistical significance when compared to siNC‐BSA. # indicates a statistical significance between siNC‐PA and siNC‐*Elk1* groups. *p *< 0.05, one‐way ANOVA

We then tested whether *Elk1* could regulate PA‐induced inflammation in 661W cells. The 661W cells were first transfected with the siRNA of *Elk1* (si*Elk1*) to knock down the ELK1 protein (Figure 4A) or a negative control (siNC) and then treated with 100 µM PA or BSA (vehicle). As PA‐treated cells had a significant increase of pP65, transfection with si*Elk1* blocked the PA‐elicited increase of pP65 (Figure 4B). The results indicate that knocking down *Elk1* alleviates PA‐induced inflammation in 661W cells.

### 3.5 Cytoplasmic versus nuclear phosphorylated ELK1 at S383 (pELK1_S383_) in retinal photoreceptors: differential effects of HFD and miR‐150 deletion in mice

**FIGURE 5:**
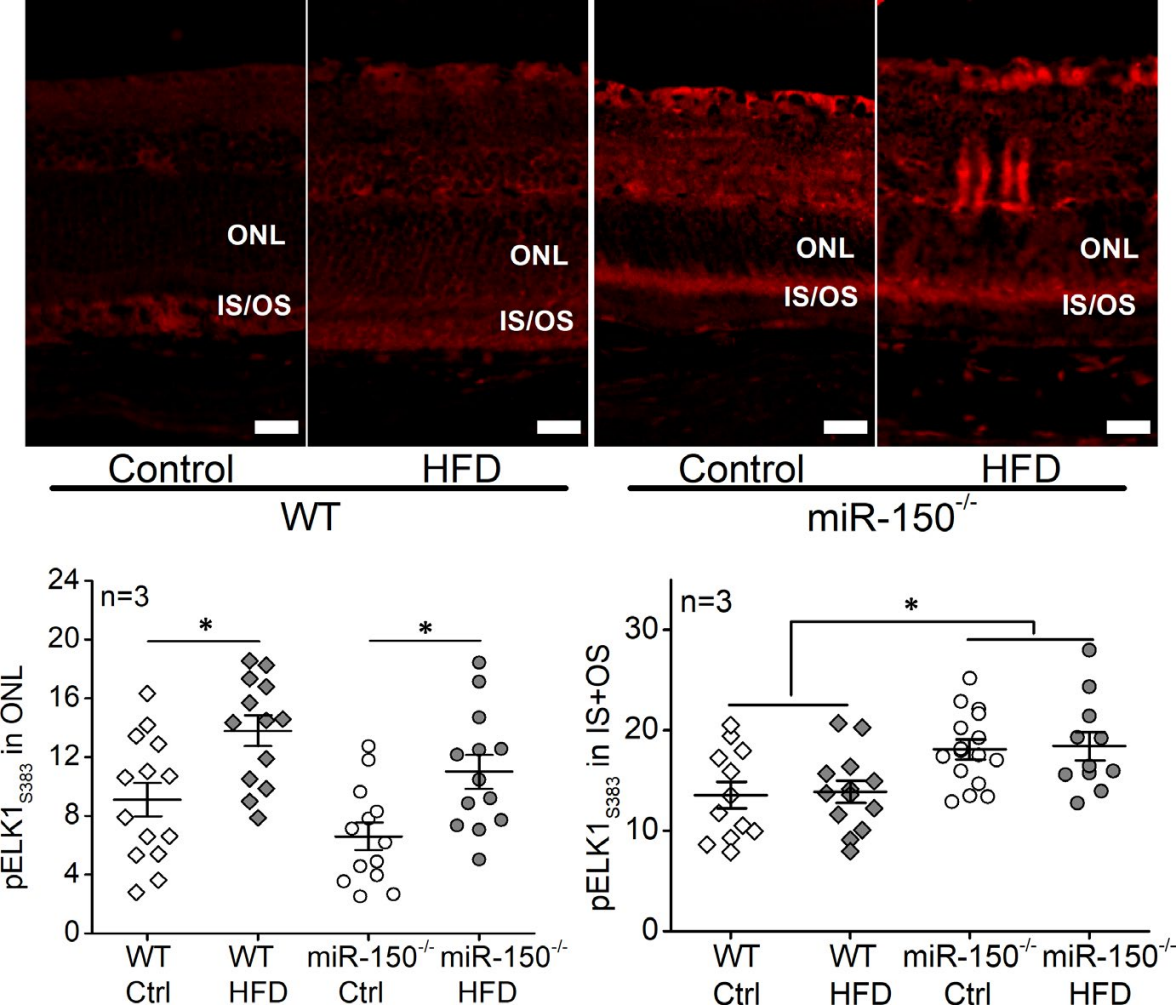
MiR‐150 knockout (miR‐150^−/−^) increases phosphorylated ELK1 at S383 (pELK1_S383_) in the inner and outer segments (IS/OS) of photoreceptors, while HFD‐induced T2D increases pELK1_S383_ in the outer nuclear layer (ONL). Six months after the diet regimens with either a normal chow (Ctrl) or HFD, the WT and miR‐150^−/−^ mouse retinas were fixed, sectioned and processed for immunostaining of pELK1_S383_ (red). The fluorescent intensities in the ONL and IS/OS were measured using ImageJ. WT‐Ctrl: open diamond; WT‐HFD: dark diamond; miR‐150^−/−^‐Ctrl: open circle; miR‐150^−/−^‐HFD: dark circle. *indicates a statistical significance between the groups specified with horizontal lines. *p *< 0.05, one‐way ANOVA. Scale bar: 20 µm

Phosphorylated ELK1 at serine 383 (pELK1_S383_) promotes inflammation as an activated transcription factor.^44^, ^45^ As we showed in Figure 1, six months after the HFD regimen, pP65 was significantly increased in retinal photoreceptors, and deletion of miR‐150 (miR‐150^−/−^) further exacerbated HFD‐induced photoreceptor inflammation. This HFD‐induced photoreceptor inflammation correlated with an increase of pELK1_S383_ in the outer nuclear layer (ONL; Figure 5). Deletion of miR‐150 (miR‐150^−/−^) significantly increased pELK1_S383_ in the inner and outer segments of photoreceptors (IS/OS) regardless of the diet regimen (Figure 5). These data imply that the HFD‐induced inflammation in photoreceptors (Figure 1) could be mediated by the increase of nuclear pELK1_S383_ (Figure 5), and the upregulated cytoplasmic pELK1_S383_ by miR‐150 knockout (Figure 5) could exacerbate the HFD‐induced inflammation (Figure 1).

### 3.6 Knocking down miR‐150 increases cytoplasmic pELK1_S383_, while PA treatments increase nuclear pELK1_S383_ in 661W cells

**FIGURE 6:**
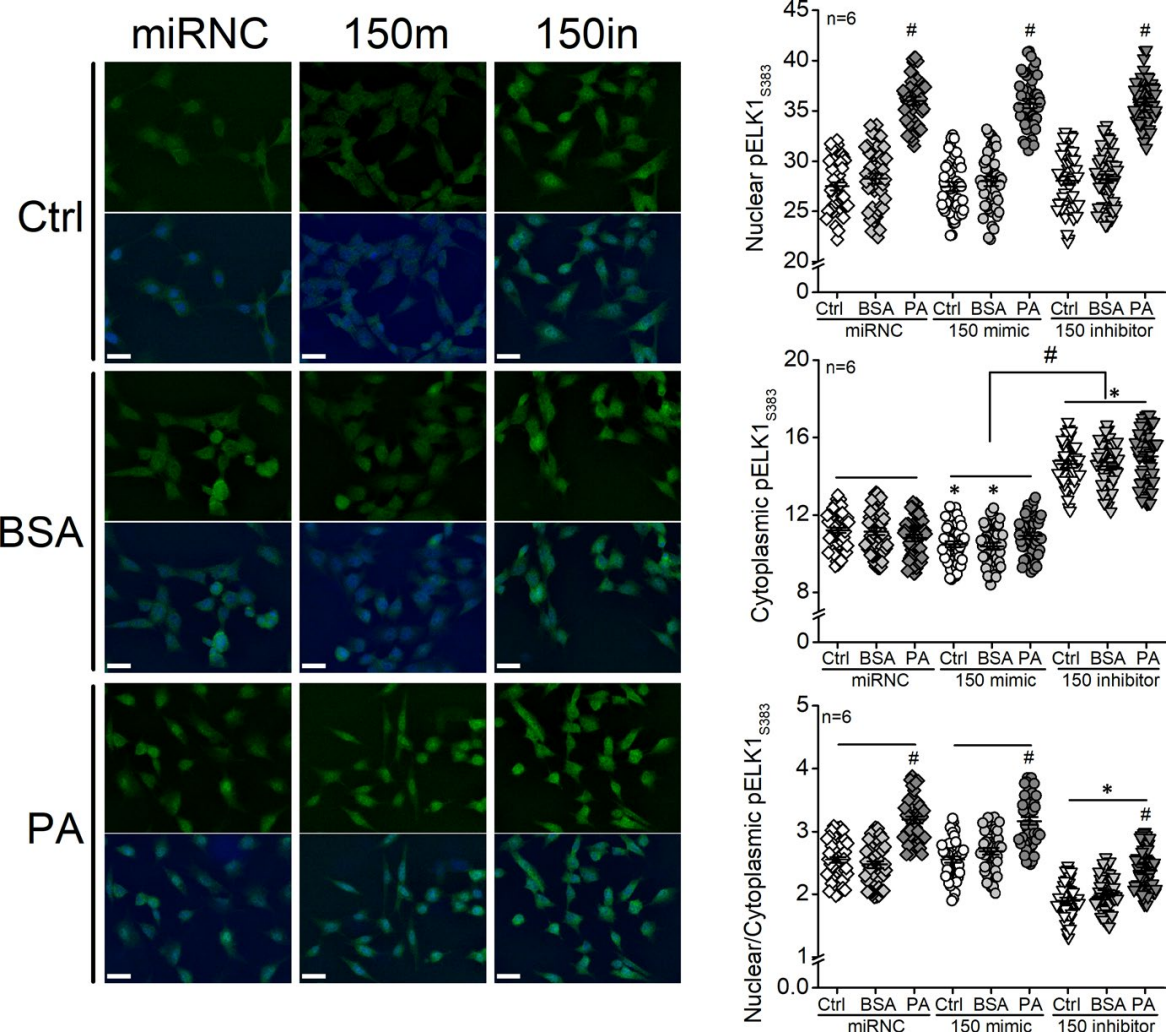
Knockdown of miR‐150 increases cytoplasmic pELK1_S383_, while PA treatments increase nuclear pELK1_S383_ in 661W cells. The 661W cells were first transfected with microRNA‐negative control (miRNC), miR‐150 mimic (150m; 150 mimic), or miR‐150 inhibitor (150in; 150 inhibitor) and then treated with culture medium (Ctrl), BSA (vehicle), or 100 µM PA (PA) for 24 h. Cells were fixed and processed for fluorescent immunostaining with pELK1_S383_ (green) and DAPI (blue). The fluorescent intensities of pELK1_S383_ in the cell nucleus and cytoplasm were measured using ImageJ, and the nuclear/cytoplasmic ratio of pELK1_S383_ was calculated. miRNC‐Ctrl: open diamond; miRNC‐BSA: grey diamond; miRNC‐PA: dark diamond; 150 mimic‐Ctrl: open circle; 150 mimic‐BSA: grey circle; 150 mimic‐PA: dark circle; 150 inhibitor‐Ctrl: open triangle; 150 inhibitor‐BSA: grey triangle; 150 inhibitor‐PA: dark triangle. Nuclear pELK1_S383_: # indicates a statistical significance when comparing PA‐treated cells to BSA‐treated cells. Cytoplasmic pELK1_S383_: * indicates that all three 150 inhibitor groups are significantly different from miRNC groups, and 150 mimic‐Ctrl and 150mimic‐BSA are also significantly different from miRNC‐Ctrl and miRNC‐BSA. # indicates that there is a statistical significance between all 150 inhibitor groups when compared to all 150 mimic groups. Nuclear/Cytoplasmic pELK1_S383_: # indicates a statistical significance when compared to the BSA groups. * indicates a statistical significance of 150 inhibitor groups when compared to the miRNC and 150 mimic groups. *p *< 0.05, one‐way ANOVA. Scale bar: 30 µm

To verify that deletion of miR‐150 further upregulated the HFD‐induced increase of pELK1_S383_, and whether overexpression of miR‐150 might prevent HFD‐induced increase of pELK1_S383_, we next examined the differential effects of PA and miR‐150 in regulating pELK1_S383_ (nucleus versus cytoplasm) in 661W cells. After 661W cells were first transfected with the miR‐150 mimic (150m; 150 mimic), miR‐150 inhibitor (150in; 150 inhibitor) or the negative control (miRNC), cells were then treated with 100 µM PA, BSA (vehicle) or culture medium (control; Ctrl) for 24 h. Treatments with PA significantly increased nuclear pELK1_S383_ regardless of the cellular levels of miR‐150 (Figure 6). Overexpression of miR‐150 (150 mimic) in BSA‐ or Ctrl‐treated cells significantly decreased cytoplasmic pELK1_S383_, while cells transfected with miR‐150 inhibitor had significant increases in cytoplasmic pELK1_S383_ regardless of their treatments (PA, BSA, or Ctrl; Figure 6). Therefore, the PA treatments increased nuclear pELK1_S383_ (Figure 6) and induced inflammation (Figure 2) in 661W cells. Knocking down miR‐150 increased cytoplasmic pELK1_S383_ (Figure 6) and promoted inflammation (Figure 2B).

Overexpression of miR‐150 (150 mimic) did not overturn the PA‐induced inflammation (Figure 2B), which may be explained by the nuclear pELK1_S383_ level of PA‐treated and miR‐150 mimic‐transfected cells (Figure 6). These data suggest that the PA‐induced increase of nuclear pELK1_S383_ (Figure 6) could mediate inflammation in 661W cells (Figure 2), and the upregulated cytoplasmic pELK1_S383_ in miR‐150 knockdown (150in) cells (Figure 6) may promote inflammation (Figure 2B). In PA‐treated cells, overexpression of miR‐150 is not sufficient to downregulate pELK1_S383_ (Figure 6) or overcome PA‐induced inflammation (Figure 2B).

### 3.7 Knocking down *Elk1* decreases cytoplasmic pELK1_S383_ and prevents PA‐elicited increase of nuclear pELK1_S383_ in 661W cells

**FIGURE 7:**
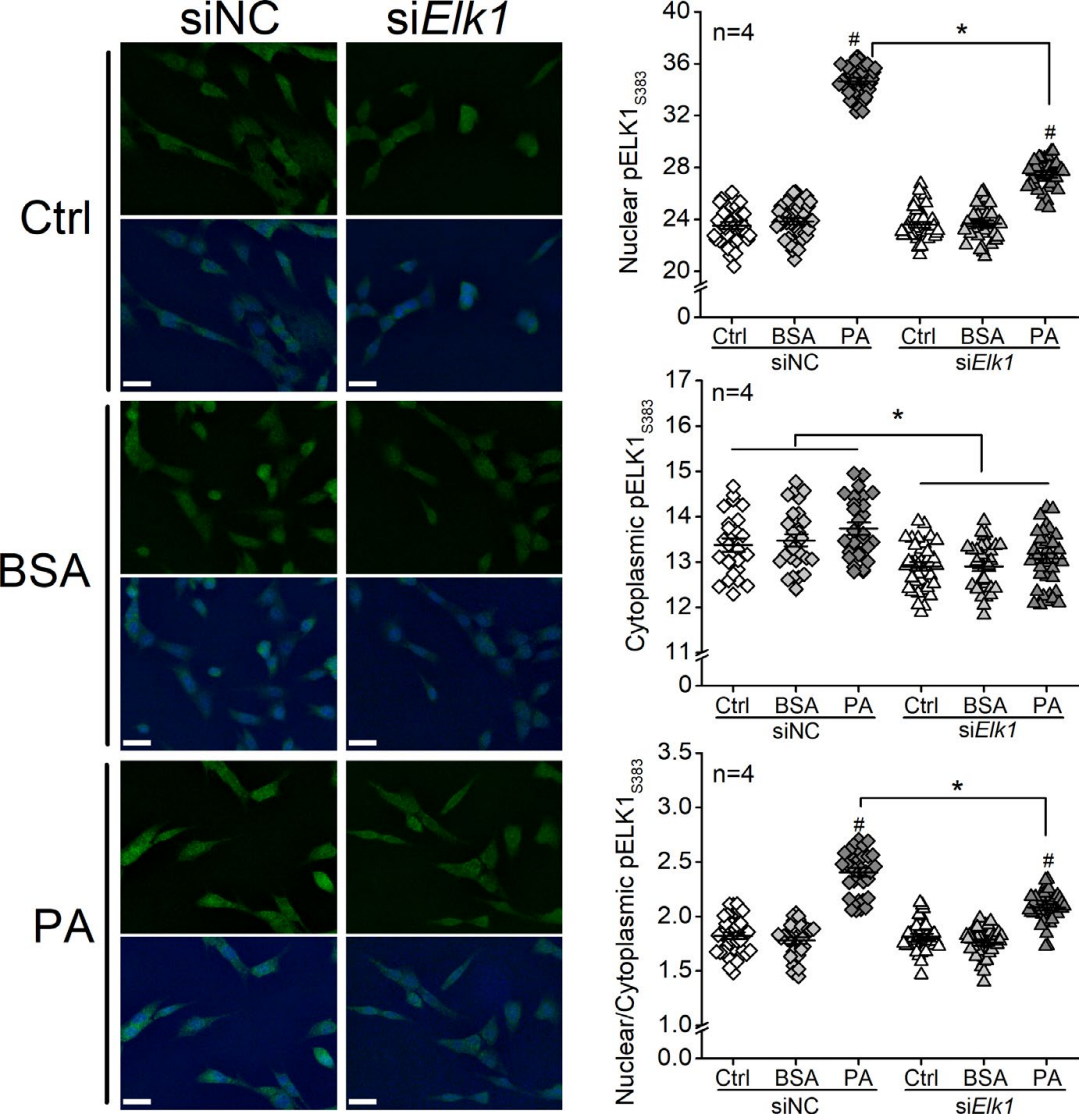
Knocking down Elk1 decreases cytoplasmic pELK1_S383_ and prevents PA‐elicited increase of nuclear pELK1_S383_ in 661W cells. 661W cells were first transfected with siRNA‐negative control (siNC) or *Elk1* siRNA (si*Elk1*) and then treated with culture medium (Ctrl), BSA or 100 µM PA (PA) for 24 h. Cells were fixed, processed, and immunostained with pELK1_S383_ (green) and DAPI (blue). The fluorescent intensities of pELK1_S383_ in the cell nucleus and cytoplasm were measured using ImageJ, and the nuclear/cytoplasmic ratio pELK1_S383_ was calculated. siRNC‐Ctrl: open diamond; siRNC‐BSA: grey diamond; siNRC‐PA: dark diamond; si*Elk1*‐Ctrl: open triangle; si*Elk1*‐BSA: grey triangle; si*ELK1*‐PA: dark triangle. Nuclear pELK1_S383_: # indicates a statistical significance when comparing PA‐treated cells to BSA‐treated cells. * indicates a statistical significance between siNC‐PA and si*Elk1*‐PA. Cytoplasmic pELK1_S383_: * indicates that all si*Elk1* groups are significantly different from all siNC groups. Nuclear/Cytoplasmic ELK1_S383_: # indicates a statistical significance when comparing PA‐treated to BSA‐treated cells. * indicates a statistical significance between siNC‐PA and si*Elk1*‐PA. *p *< 0.05, one‐way ANOVA. Scale bar: 30 µm

We showed that knocking down *Elk1* alleviated PA‐induced inflammation in 661W cells (Figure 4). In order to understand whether the suppression of inflammation is correlated with the cellular distribution of pELK1_S383_, we used siRNA to knock down *Elk1 (*si*Elk1)* to determine cytoplasmic versus nuclear pELK1_S383_ levels in 661W cells. While PA treatments elicited significant increases of nuclear pELK1_S383_, transfection with si*Elk1* significantly decreased cytoplasmic pELK1_S383_ and attenuated the PA‐induced increase of nuclear pELK1_S383_ compared to cells transfected with the negative control (siNC; Figure 7). Therefore, knocking down *Elk1* decreased PA‐elicited increases of pELK1_S383_ (Figure 7), which implies that downregulation of pELK1_S383_ could dampen PA‐induced inflammation (Figure 4).

## 4 DISCUSSION

Chronic inflammation is a manifestation of diabetic retinas.^23^, ^40^, ^46^ Diabetic conditions such as hyperlipidaemia and hyperglycaemia cause increases of pro‐inflammatory molecules in retinal neurons including photoreceptors.^47^, ^48^ Photoreceptors are major contributors to inflammation in the diabetic retina,^11^, ^15^ but we still do not have a complete picture of how diabetic conditions elicit inflammation in photoreceptors. MiR‐150 is a suppressor of inflammation.^19^, ^31^, ^49^ Under diabetic insults, deletion of miR‐150 upregulated the levels of pro‐inflammatory cytokines,^19^, ^23^ while miR‐150 overexpression reduced the expression of those molecules.^50^ We found that pP65, a biomarker of inflammation, increased in both inner and outer segments and the ONL of photoreceptors in the mouse retina under HFD‐induced T2D, and deletion of miR‐150 further exacerbated the HFD‐induced increase of pP65 (Figure 1). Others have shown that overexpression of miR‐150 suppresses pP65 by downregulating the target genes of miR‐150.

Using cultured 661W cells, we identified that *Elk1* is the direct target of miR‐150 that mediates PA‐elicited inflammation. The activation and specific function of ELK1 are phosphorylation site‐dependent,^51^, ^52^ as phosphorylation on S383 (pELK1_S383_) is known to promote inflammation.^44^, ^45^ Phosphorylation site‐dependent functions for a protein are not unique to ELK1. For example, phosphorylation of AMP‐activated protein kinase (AMPK) at Ser485/491 facilitates cardiac hypertrophy, while phosphorylated AMPK at Thr172 mediates the antihypertrophic response.^53^ Phosphorylated ELK1 at S383 (pELK1_S383_) translocates from the cytoplasm to the nucleus, and nuclear pELK1_S383_ further transactivates its downstream genes to promote inflammation.^44^, ^45^ In this study, we found that pELK1_S383_ increased in the retinal ONL of the HFD mice (Figure 5) and the nuclei of PA‐treated 661W cells (Figure 6), and the increased nuclear pELK1_S383_ correlates with the upregulated pP65 in photoreceptors under the HFD regimen or PA treatments (HFD/PA). Knocking out miR‐150 further upregulated cytoplasmic pELK1_S383_ as well as exacerbated HFD/PA‐elicited increase of pP65 (Figures 1 and 2). Our results suggest that HFD/PA may induce inflammation in photoreceptors by increasing nuclear pELK1_S383_, and the upregulated cytoplasmic pELK1_S383_ could further exacerbate the inflammation. While phosphorylated ELK1 promotes the transcription of downstream genes, SUMOylated ELK1 represses the transactivation activity.^54^, ^55^ Stress elevates the phosphorylation of ELK1 but removing the SUMOylation.^56^, ^57^ It is possible that decreased SUMOylated ELK1 in the cytoplasm partially contributes to the upregulation of cytoplasmic pELK1_S383_ (Figures 5 and 6), which may explain the exacerbated inflammation in miR‐150‐knockdown cells.

The nuclear content of pELK1_S383_ relative to that in the cytoplasm (nuclear/cytoplasmic ratio; N/C) represents the translocation of pELK1_S383_ from the cytoplasm to the nucleus, in which an increased N/C value of pELK1_S383_ implies an increased inflammation at the cellular level.^44^, ^45^ We showed that PA treatments increased the N/C value of pELK1_S383_ in 661W cells (Figure 6), and knocking down *Elk1* (si*Elk1*) decreased nuclear pELK1_S383_ and the N/C value in PA‐treated 661W cells (Figure 7), which correlated with the downregulation of pP65 (Figure 4B). In addition, cytoplasmic pELK1_S383_ was decreased in the si*Elk1* cells, which may further repress the activity of ELK1. The results imply that knocking down *Elk1* can potentially alleviate diabetes‐associated inflammation in retinal photoreceptors.

Inflammation of retinal photoreceptors in diabetes correlates with dampened light responses in the retina.^23^, ^40^ Although pP65 is not significantly downregulated by overexpression of miR‐150 (Figure 2B), other pro‐inflammatory molecules such as TNF‐α are diminished by overexpression of miR‐150.^31^, ^49^ Moreover, miR‐150 can reduce the apoptosis of endothelial cells under inflammation.^31^ Therefore, overexpressing miR‐150 could protect retinal endothelial cells from diabetic insults. In this study, knocking down *Elk1* reduces pP65 in PA‐treated 661W cells, indicating that pP65 is regulated by ELK1, although NFĸB is not a direct target of ELK1. Since inhibiting NFĸB P65 alleviates high glucose‐induced apoptosis in endothelial cells,^58^, ^59^ knockdown of *Elk1* could mitigate diabetes‐induced damage to the retinal vasculature. Therefore, *Elk1* might be a new target for future therapeutics to treat diabetes‐associated inflammation and potentially prevent the development of DR.

## CONFLICT OF INTEREST

The authors confirm that there are no conflicts of interest.

## AUTHOR CONTRIBUTION

**Fei Yu:** Conceptualization (equal); Data curation (equal); Formal analysis (equal); Methodology (equal); Validation (equal); Writing‐original draft (equal). **Michael L. Ko:** Methodology (equal); Project administration (equal); Writing‐review & editing (equal). **Gladys Y.‐P. Ko:** Conceptualization (equal); Funding acquisition (equal); Investigation (equal); Resources (equal); Supervision (equal); Writing‐original draft (equal); Writing‐review & editing (equal).

## Data Availability

The data presented in this study are available from Fei Yu and Dr. Gladys Ko upon reasonable request.
